# Ecogroups and maternal haplogroups reveal the ancestral origin of native Chinese goat populations based on the variation of mtDNA D‐loop sequences

**DOI:** 10.1002/ece3.10382

**Published:** 2023-08-07

**Authors:** Wenjuan Qin, Daosong Chen, Panpan Guo, Lixing Hu, Xiaodong Zheng, Jin Cheng, Hongquan Chen

**Affiliations:** ^1^ School of Animal Science and Technology Anhui Agricultural University Hefei China; ^2^ Key Laboratory of Anhui Local Livestock and Poultry Genetic Resources Conservation and Biobreeding of Anhui Province Hefei China; ^3^ Animal Molecular Immunization Center of Anhui Agricultural University Hefei China; ^4^ Hefei Institutes of Physical Science Chinese Academy of Sciences Hefei China; ^5^ Department of Dermatology The First Affiliated Hospital of Anhui Medical University Hefei China; ^6^ College of Biological and Pharmaceutical Engineering West Anhui University Luan China

**Keywords:** conservation biology, ecogroup, genetic differentiation, maternal origin, molecular evolution, population expansion

## Abstract

China is rich in goat breeding resources. Officially recognized local goat breeds are mainly distributed in agro‐ecological regions. The population structure and matrilineal origin of native Chinese goats can be used to formulate protection and utilization strategies for these genetic resources. In this study, the genetic structure and maternal origin of native Chinese goats were investigated using mtDNA D‐loop sequences. A total of 329 goat samples from 25 Chinese indigenous goat populations and five introduced goat breeds from abroad were collected; these populations were distributed in four ecogroups designated as Southwest, South‐central, the North China Plain, and Foreign‐ecogroup. A larger average number of nucleotide differences and richer nucleotide diversity were observed in South‐central and Foreign‐ecogroup, whereas these were lower in Southwest. The 216 haplotypes divided into several haplogroups, of which HapA contained 99 haplotypes distributed in Southwest, the North China Plain, and Foreign‐ecogroup with high frequency (0.53–0.77), whereas the frequency of HapA in South‐central was <0.09. HapB was mostly found in South‐central (0.5538) and was distributed to the North China Plain (0.2667), while it was rare in Southwest (<0.08) and Foreign‐ecogroup (<0.07). According to the estimation of kinship and ancestry, HapA had five ancestors (A2, A3, A5, A10, and A12), HapB had a single maternal ancestor (A8), and HapC had two maternal ancestors (A1 and A4). This study showed that native Chinese goat breeds were mainly divided into three haplogroups (HapA, HapB, and HapC) and goat populations have expanded in the ecological regions.

## Graphical abstract

Native Chinese goat breeds were mainly divided into three haplogroups: HapA, HapB, and HapC, originated from five, one, and two maternal ancestors, respectively, and were associated with the ecogroups. The goat populations expanded in the different ecoregions. The relative evolutionary rate of goat mtDNA D‐loop differed among the ecogroups.

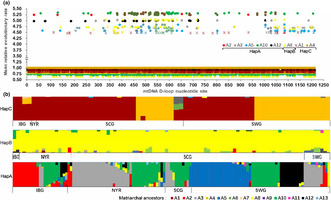

## 1 INTRODUCTION

**FIGURE 1:**
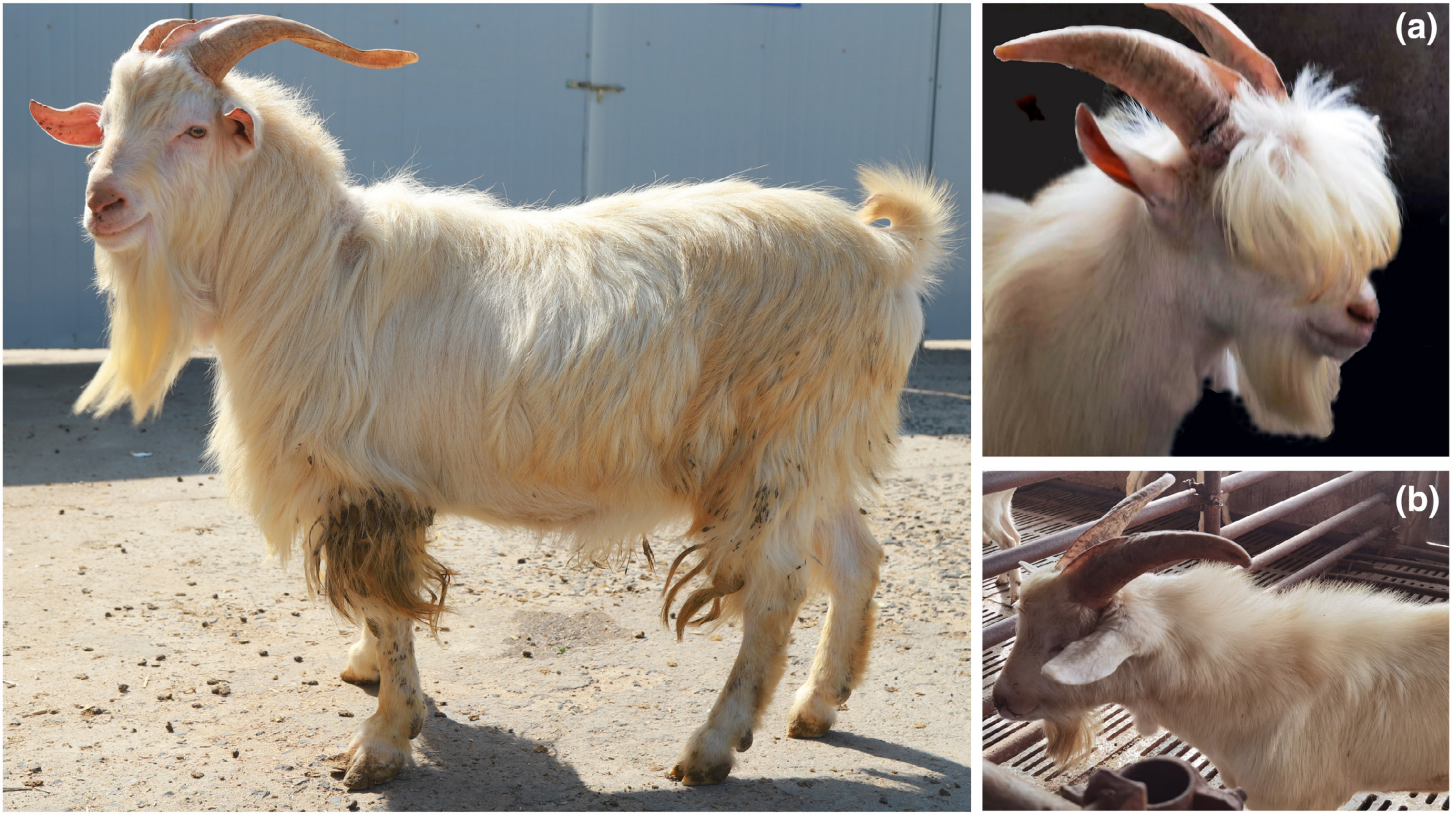
Qianqiu goat (QQ goat). (a) The blossoming long hair on the forehead of QQ; (b) The erect long hair on the neck and shoulders of QQ.

China has rich goat resources, including officially recognized local and cosmopolitan breeds, and has a long breeding history (Tu et al., 1989). A study suggested that the common ancestor of Chinese goats originated in western Iran, and modern Chinese goat breeds have differentiated into two large groups that adapt to dry cold environments in the north and humid hot environments in the south (Cai et al., 2020). In addition, some ancient indigenous goat populations were identified in the third livestock and poultry genetic resources survey in China. For example, Qianqiu goats (QQ, Figure 1), which originated in Tianchang City, China, have been reared for more than 1270 years. During this time period, there was a legal system that governed growing grass and rearing sheep, and people paid taxes with sheep skins (Xia et al., 1992). However, the evolutionary history and migration routes of these Chinese goat breeds remain poorly understood. In China, goat breeding for mutton, milk, sheepskin, and cashmere has a long tradition as well as economic importance for providing Chinese people with winter food and warm clothing. China is comprised of complex terrain and varying climate with frigid, temperate, and torrid zones, and nearly 50 Chinese indigenous goat breeds (Tu et al., 1989). According to the list of Chinese breed resources, there are more than 70 goat breeds in China (National Animal Husbandry Station (China), 2022). The supply of goat meat has increased in China with consumer demand. In the 1970s, China introduced foreign goat breeds to improve the carcass and lactation performance of local goats, which enriched the genetic basis of native goats in China but also brought severe challenges to the protection of indigenous Chinese goat breeds (Chen et al., 2012; Jin et al., 2012). Therefore, it is important to identify and protect the genetic resources of goats in China. The limited information available on the characteristics of indigenous Chinese goats is mostly based on phenotypic features, which can be subjective and dependent on the environment, making it difficult to distinguish between populations (Falconer & Mackay, 1996).

**FIGURE 2:**
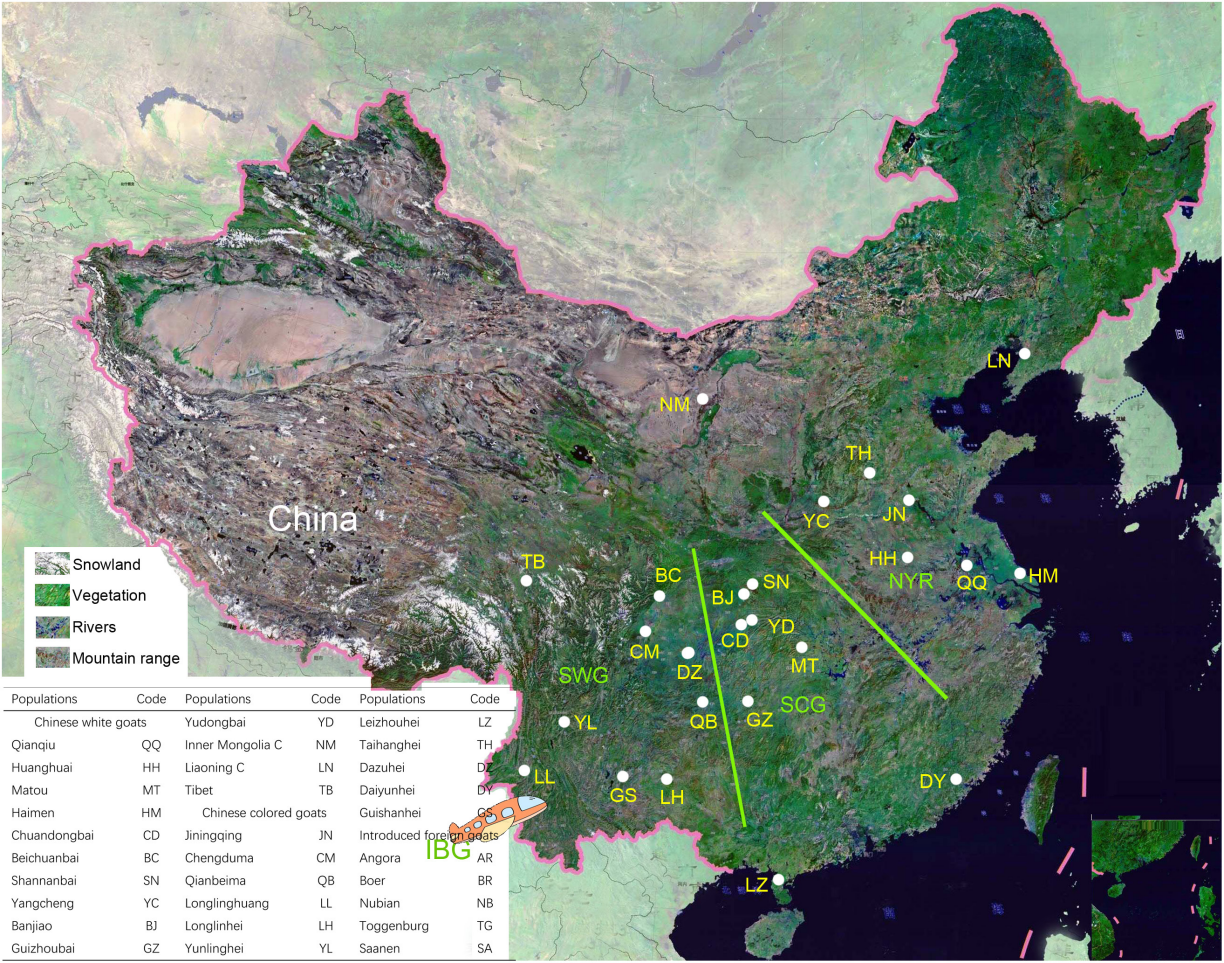
Distribution of 25 Chinese native goat populations. Geographical agro‐ecological regions defined as the southwest (SWG), South‐central (SCG) and the North China Plain to Yangtze River (NYR); IBG, Introduced foreign goats.

The majority of native goat breeds in China are distributed in the southwest plateau (SWG), south‐central mountains and hills (SCG), and North China Plain to Yangtze River (NYR) regions (Figure 2). The barrier effects of rivers and mountains have led to the formation of many ecological groups (ecogroups) in Chinese goat populations (Chen et al., 2012). However, the real origins of native Chinese goats remain unexplored and enigmatic (Wang et al., 2017; Wei et al., 2014). Little is known about how and when local goats inhabited their domestic regions, the phylogenetic relationships within and among populations, and their migration routes associated with important social and cultural characteristics.

Polymorphisms of mitochondrial DNA (mtDNA) are widely used to study the origin, maternal lineages, and domestication processes, and to clarify the migration routes of goat populations (Al‐Araimi et al., 2017), which were reflected in the complete mitogenomics (Doro et al., 2014; Piras et al., 2012; Zhang et al., 2016). Additionally, this will help to construct the populations with nuclear families or rare haplotypes for specific exploitable aims in the maternal genetic characteristics (Deniskova et al., 2020), and to elucidate the impact of introduced goats on local goat genetic resources, providing a basis for the protection of indigenous goat breeds in China. In this study, we aimed to assess the genetic diversities and to determine the maternal origins and population history of indigenous Chinese goat populations by analyzing D‐loop mtDNA sequences.

## 2 MATERIALS AND METHODS

### 2.1 Sample collection

A total of 329 samples were collected from 30 goat breeds, of which this included 299 unrelated goats from 25 indigenous goat populations in major ecoregions and 30 goats from five introduced foreign goat populations. All goat populations were divided into four ecogroups: SWG, SCG, NYR, and IBG (Table 1). To establish the genetic relationships of the studied goat populations with goats from different geographical locations, we included an additional 300 mtDNA D‐loop sequences from the NCBI database (Table S1).

**TABLE 1:** The samples of goat populations and their geographical agro‐ecological groups.

Populations	*N*	EG	Populations	*N*	EG	Populations	*N*	EG
Chinese white goat			Yudongbai	9	SCG	Chengduma	20	SWG
Qianqiu	29	NYR	Chuandongbai	5	SCG	Dazuhei	14	SWG
Huanghuai	14	NYR	Beichuanbai	4	SWG	Guishanhei	12	SWG
Yangcheng	4	NYR	Tibet	9	SWG	Yunlinghei	12	SGW
Liaoning C	5	NYR	Chinese colored goat			Longlinhei	14	SWG
Inner Mongolia C	1	NYR	Taihanghei	5	NYR	Introduced foreign goat		
Haimen	1	NYR	Jiningqing	1	NYR	Angora	5	IBG
Banjiao	29	SCG	Leizhouhei	27	SCG	Boer	4	IBG
Shannanbai	16	SCG	Daiyunhei	15	SCG	Nubian	3	IBG
Matou	14	SCG	Qianbeima	10	SWG	Toggenburg	7	IBG
Guizhoubai	15	SCG	Longlinghuang	14	SWG	Saanen	11	IBG

Abbreviations: EG, ecogroup; *N*, sample number; NYR, northern group of the Yangtze River (North China Plain); SCG, south‐central group; SWG, southwest group.

Qianqiu (QQ) is a newly discovered and professionally recognized indigenous goat population. The 29 QQ samples of whole blood from the jugular vein were collected by trained personnel under strict veterinary rules in accordance with the relevant officially registered breeding farms. This study was performed in accordance with the ethical guidelines of the Anhui Society of Animal Husbandry and Veterinary Medicine. We selected phenotypically typical animals. Only unrelated goats were used in this study. The 300 samples from the other 29 populations were from officially recognized goat breeds, and the mtDNA D‐loop sequence information was seen in Table S1.

### 2.2 PCR amplification, purification, and sequencing

Genomic DNA was extracted from whole blood using commercial DNA‐Extran‐2 kits (CJSC Syntol), according to the manufacturer's instructions. The mtDNA D‐loop sequence, which was obtained from the NCBI database (GenBank accession number AF533441), was used as a reference to design primers (forward: 5′‐ CCCTAAGAGTCAAGGAAGAAGCC ‐3′ and reverse: 5′‐ GTGTGCTTGATACCTGCTCCTCT ‐3′) to amplify the 1437‐bp D‐loop region. Primers were synthesized by General Biology Co., Ltd. The PCR reaction volume was 50 μL, which included 1 μL of DNA template (75 ng/μL), 25 μL 2× Hieff PCR Master Mix, 2 μL of each primer (10 μmol/L), and 20 μL of ddH_2_O. Amplification was performed using a TProfessional Standard Gradient Thermocycler (Biometra) under the following conditions: 94°C for 5 min; 22 cycles of 94°C for 30 s, 55°C for 30 s, and 72°C for 90 s; and a final extension at 72°C for 10 min. The quality of PCR products was evaluated using 1% agarose gel electrophoresis with ethidium bromide (0.5 μg/mL).

E‐Gel CloneWell II agarose gels (Thermo Fisher) were used to purify the PCR products. Purified PCR products were submitted to General Biology (Anhui) for Sanger sequencing from both ends.

### 2.3 Sequence alignment and data analysis

The nucleotide sequences were edited and aligned using DNAStar EditSeq and MEGA version 11 (Tamura et al., 2021) software. DnaSP version 6 was used to calculate the haplotype number, haplotype diversity, nucleotide diversity, variable site number, and average number of nucleotide differences (Rozas et al., 2017). To determine the source of genetic variation among populations, AMOVA, mismatch distribution, and neutrality tests were performed using Arlequin version 3.5 (Excoffier & Lischer, 2010).

BIONJ was used to construct the phylogenetic tree using all generated sequences with 1000 bootstrap iterations (Gascuel, 1997). Haplotypes were determined using DnaSP version 6 software (Librado & Rozas, 2009), and median‐joining networks for various haplotypes were constructed using PopART version 4 software (French et al., 2014). The degree of differentiation and population structure of the investigated breeds were assessed using ADMIXTURE version 1.3 software (Alexander et al., 2009). A good value of *K* exhibits a low cross‐validation error compared with other *K* values (*K* = 2–15).

The MER per site of the mtDNA D‐loop sequences was estimated using the Tamura and Nei (1993) model (+G). These rates were scaled such that the average evolutionary rate across all the sites was 1. This means that sites showing a rate <1 are evolving slower than the average, and those with a rate >1 are evolving faster than the average. In addition, a haplotype profile was constructed using the haplogroup distribution in the population. The proportion of haplogroups in the population was used to create the haplogroup component diagram.

## 3 RESULTS

### 3.1 Genetic diversity and evolutionary rate of D‐loop MtDNA

According to the sequencing results, the sequence lengths of D‐loop mtDNA in Qianqiu goats were 1212 bp and 1213 bp, accounting for 72.41% and 27.59% in the population, respectively. The latter was caused by the insertion of a base C at position 1068. The D‐loop sequences of 329 individuals from 30 goat populations were compared in Appendix S1; a total of 246 nucleotide substitution sites were detected, of which 93 were singleton variable sites and 153 were parsimony‐informative sites. In addition, the polymorphic sites defined 216 haplotypes in 329 individuals, of which 174 haplotypes were unique. Hap22 was shared by 12 individuals from BJ, DZ, GS, GZ, LZ, and SN; Hap56 was shared by 12 individuals from DY, GZ, and LZ; Hap23 by 11 individuals from BJ, CD, GZ, and MT; and Hap44 was shared by 11 individuals from CM, GZ, LH, and LL.

The haplotypes and genetic diversity of the four ecogroups are shown in Table 2. Nucleotide diversity did not significantly differ between white goats and colored goats, *π* = 0.0393 vs. 0.0357, and *K* = 47.606 vs. 43.085, respectively. However, there was a sharp difference between the ecogroups; the average number of nucleotide differences and diversity in SCG (57.009 and 0.0471) and IBG (54.389 and 0.0451) were greater than those in SWG (24.837 and 0.0205). Haplotype diversity was between 0.9870 and 0.9955 for all ecogroups and colored and white goats, respectively.

**TABLE 2:** Genetic diversity of mitochondrial DNA (mtDNA) in the goats reared in China.

	*B*	*N*	*S*	*H*	Hd	*K*	*π*
Goat group							
White goat	17	175	249	138	0.9955 ± 0.0015	47.606	0.0393 ± 0.0031
Colored goat	13	154	250	104	0.9879 ± 0.0033	43.085	0.0357 ± 0.0029
Ecogroups							
SWG	9	109	157	76	0.9870 ± 0.0050	24.837	0.0205 ± 0.0009
SCG	8	130	198	88	0.9880 ± 0.0030	57.009	0.0471 ± 0.0026
NYR	8	60	216	49	0.9900 ± 0.0060	36.519	0.0302 ± 0.0054
IBG	5	30	202	28	0.9950 ± 0.0100	54.389	0.0451 ± 0.0074
All populations	30	329	267	216	0.9930 ± 0.0010	20.574	0.0170 ± 0.0006

Abbreviations: *B*, population (breed) number; *H*, number of haplotypes; HD, haplotype diversity; *K*, average number of nucleotide differences; *N*, sample number; *S*, number of variable sites; *π*, nucleotide diversity.

**FIGURE 3:**
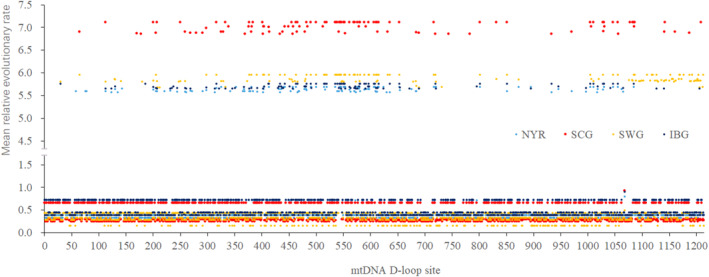
The mean (relative) evolutionary rate per sites of mtDNA D‐loop of 329 goat individuals. These rates are scaled such that the average evolutionary rate across all sites is 1. This means that sites showing a rate <1 are evolving slower than average, and those with a rate >1 are evolving faster than average. These relative rate were estimated under the Tamura and Nei (1993) model (+G). NYR, SCG, SWG, and IBG were the ecogroups defined in Figure 2.

The nucleotide substitution frequencies in the D‐loop sequences were analyzed to identify, high‐frequency site regions (HFSR). From Figure 3, the results showed that these sites were clustered in 300–600 bp and 1000–1200 bp sequences, with a higher mean relative evolutionary rate (MER) for each site. The MERs of the HFSR evolved faster than average, and those of the SCG evolved faster than other ecogoups.

### 3.2 Maternal haplogroup

**FIGURE 4:**
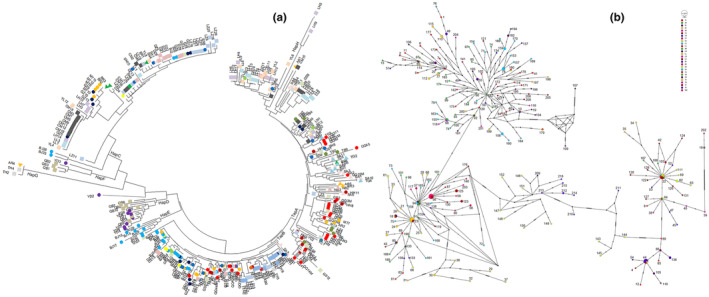
The phylogenetic tree and distance to the ancestor of goat D‐loop mtDNA for 329 goat individuals. (a) ○ white Chinese goats, □ colored Chinese goats, and △ introduced goats were marked different colors. (b) Median‐joining networks for the 216 various haplotypes found in 329 individuals from 30 populations. Each link between haplotypes represents one mutational difference. The labeled nodes with number indicate Haplotype while unlabeled nodes indicate inferred steps not found in the sampled populations.

Phylogenetic analysis was conducted to assess the relationship between the ecogroups and populations and their respective maternal origins. The 329 sequences were used to construct the neighbor‐joining (BIONJ) tree and median‐joining (MJ) network (Figure 4). Both the tree in Figure 4a and MJ network in Figure 4b showed that all goats were classified into distinct groups, which represented haplogroups A (HapA), B, and C, containing 99, 52, and 37 haplotypes, respectively.

**FIGURE 5:**
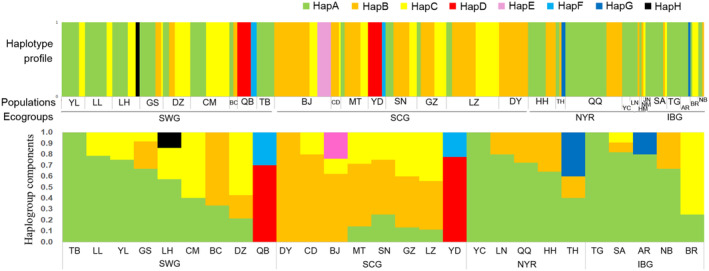
Haplotype profile of goat breeds in different ecological regions. The width of the profile indicated the haplotype type (haplogroup) and its sample size. Haplogroup components were the proportion of haplogroups within the population. HapB was the dominant haplotypes for SCG, HapA for SWG, NYR, and IBG, and HapD for QB and YD populations.

The haplotype profile was constructed using the haplogroup distributions of the different ecogroup populations in Figure 5. HapA was the dominant haplotype in SWG (0.5321), NYR (0.6833), and IBG (0.7667), whereas HapA was rare in SCG (0.0846). HapB was mostly found in SCG (0.5538), followed by NYR (0.2667); however, this haplotype was rare in SWG (0.0734) and IBG (0.0667). HapC was distributed in the SWG and SCG at a medium frequency (0.2844 and 0.2385, respectively). HapD and HapF were only observed in the QB and YD populations.

### 3.3 Population structure and matriarchal ancestries

The body‐color and habitats of goats were compared by AMOVA (Table 3) which revealed that a significant (*p* < .01) proportion of the total genetic variation occurred among the goat ecogroups (7.95%), among breeds within ecogroups (15.35%), and within the goat breeds (76.70%). The variation between the body‐color groups accounted for only 2.29% (*p* > .05), while the variation between breeds within body‐color groups accounted for 20.17% (*p* < .01) and within breeds was 77.54% (*p* < .01). These results suggest that diversity is related to the geographical distribution of the goat populations.

**TABLE 3:** AMOVA based on the analysis of the complete mtDNA D‐loop.

Source of variation	df	Sum of squares	Variance	% of variation	*p* value
Body‐color grouping					
Among groups	1	82.013	0.240	2.29	.083
Among breeds within groups	26	839.983	2.120	20.17	.000**
Within breeds	301	2452.117	8.147	77.54	.000**
Total	328	3374.113	10.494		
Ecology grouping					
Among ecogroups	3	286.604	0.844	7.95	.001**
Among breeds within ecogroups	24	635.392	1.630	15.35	.000**
Within breeds	301	2452.117	8.147	76.7	.000**
Total	328	3374.113	10.656		

*Note*: Statistically significant, **p < .01; Body‐color grouping: colored goat breed and white goat breed groups; ecology grouping: SWG, SCG, NYR, and IBG ecogroups.

Abbreviation: df, degrees of freedom.

**FIGURE 6:**
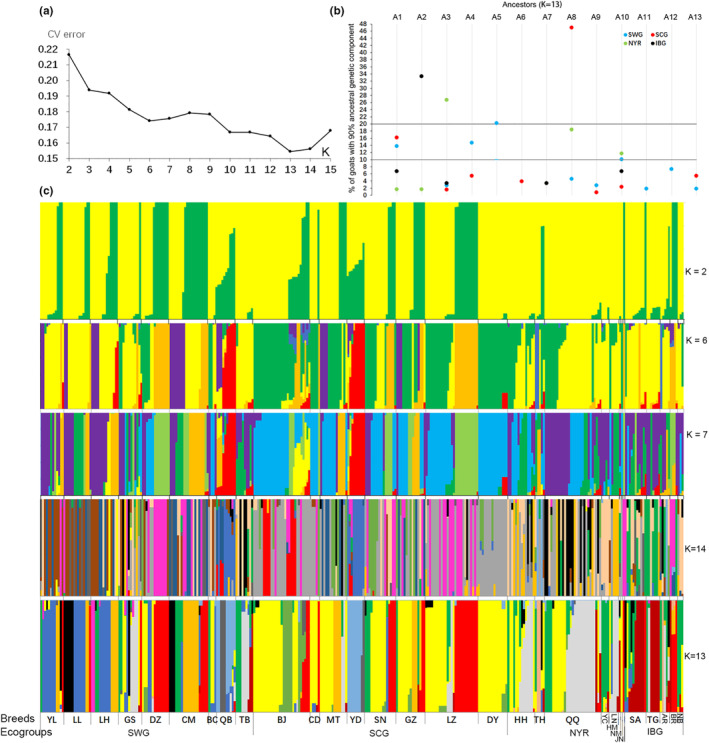
The Population structure of 30 goat breeds. (a) estimating CV error with K ancestry clusters; (b) % of goats with more than 90% of ancestral genetic components; (c) Estimated population structure of the analyzed breeds represented as bar plot depicting individual membership proportions for each cluster. The codes of breeds and ecogroups were showed in Table 1 and Figure 2, respectively.

The matriarchal ancestries of the goat breeds were estimated using the ADMIXTURE program, with *K* expected clusters ranging from 2 to 15. The optimal number of ancestral populations with the lowest CV error was *K* = 13 (Figure 6a). At *K* = 6 or 7, the distinct classification of goat breeds only remained at the ecogroup level. At *K* = 13, the maternal ancestors among the breeds became clear (Figure 6c). The ratio (RDA) of goats with more than 90% ancestral components (dominant ancestors) was calculated in Figure 6b. The top four dominant ancestors were A8 for SCG (46.92%), A2 for IBG (33.33%), A3 for NYR (26.67%), and A5 for SWG (20.18%), respectively. This was followed by A8 for NYR (18.33%), A1 for SCG (16.15%) and SWG (13.76%), A4 for SWG (14.68%), and A10 for SWG (10.09%) and NYR (11.67%). These results indicate that Chinese native goats in different ecoregions originated from two to three matrilineal ancestors and had no homology with imported goats.

**FIGURE 7:**
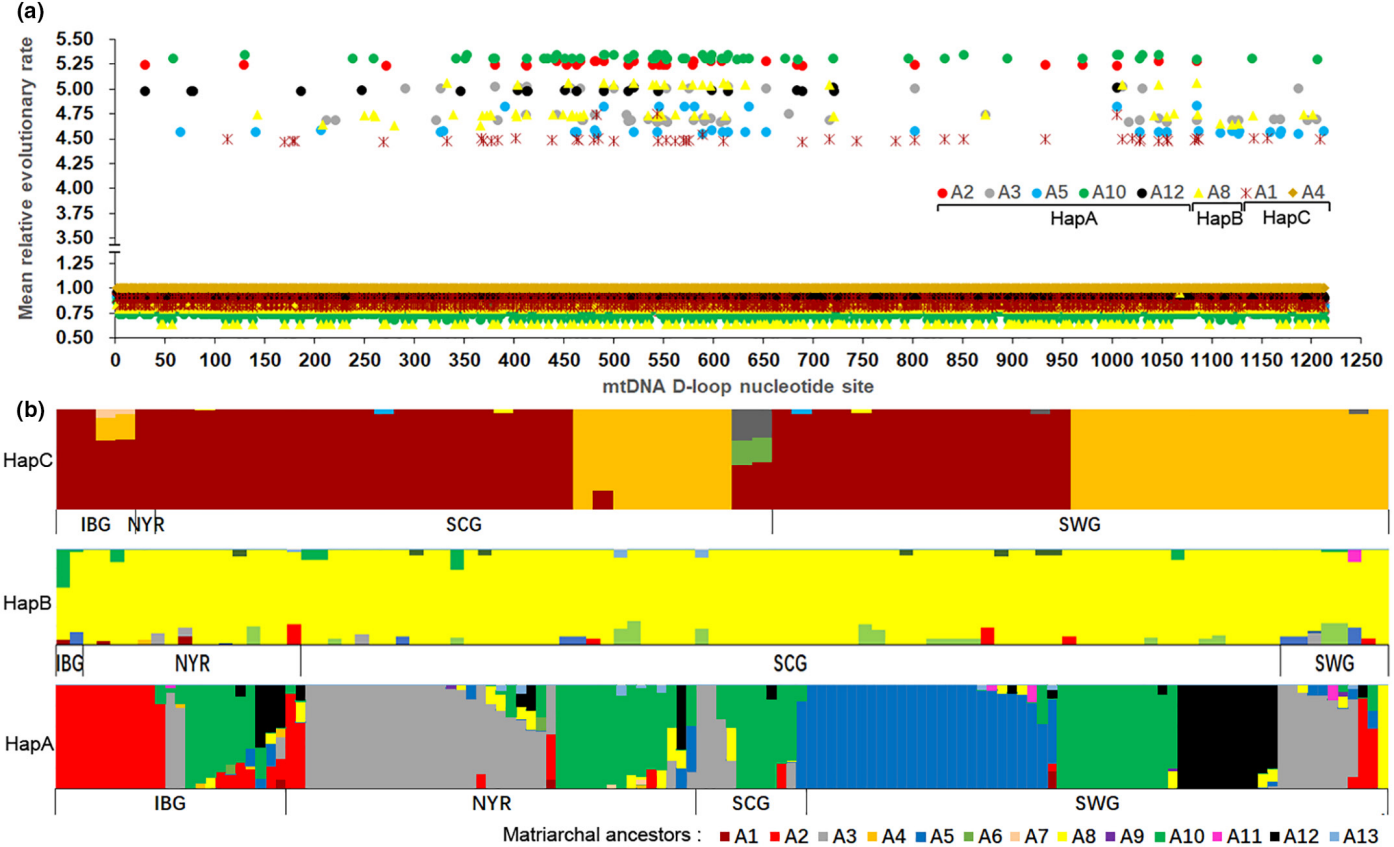
The maternal ancestors of three Haplogroups (b) and the mean relative evolutionary rate of each sites in the ancestor mtDNA D‐loop (a). SWG, SCG, NYR, and IBG were ecogroups and defined in Figure 2. A2, A3, A5, A10 and A12 were the ancestors of HapA, A8 was the ancestor of HapB, and A1 and A4 were the ancestors of HapC (b).

HapA was associated with five ancestors (A2, A3, A5, A10, and A12), HapB was associated with a single maternal ancestor (A8), and HapC was associated with two ancestors (A1 and A4; Figure 7b). the MER of each site in the mtDNA D‐loop sequence of different maternal ancestors was estimated (Figure 7a) and found that the MER of HFSR of ancestors A2 and A10 were higher than those of A1, A5, and A4 and that the MER of HFSR of A8 was between these two groups of ancestors.

### 3.4 Population demographic history based on the ecogroups and haplogroups

**FIGURE 8:**
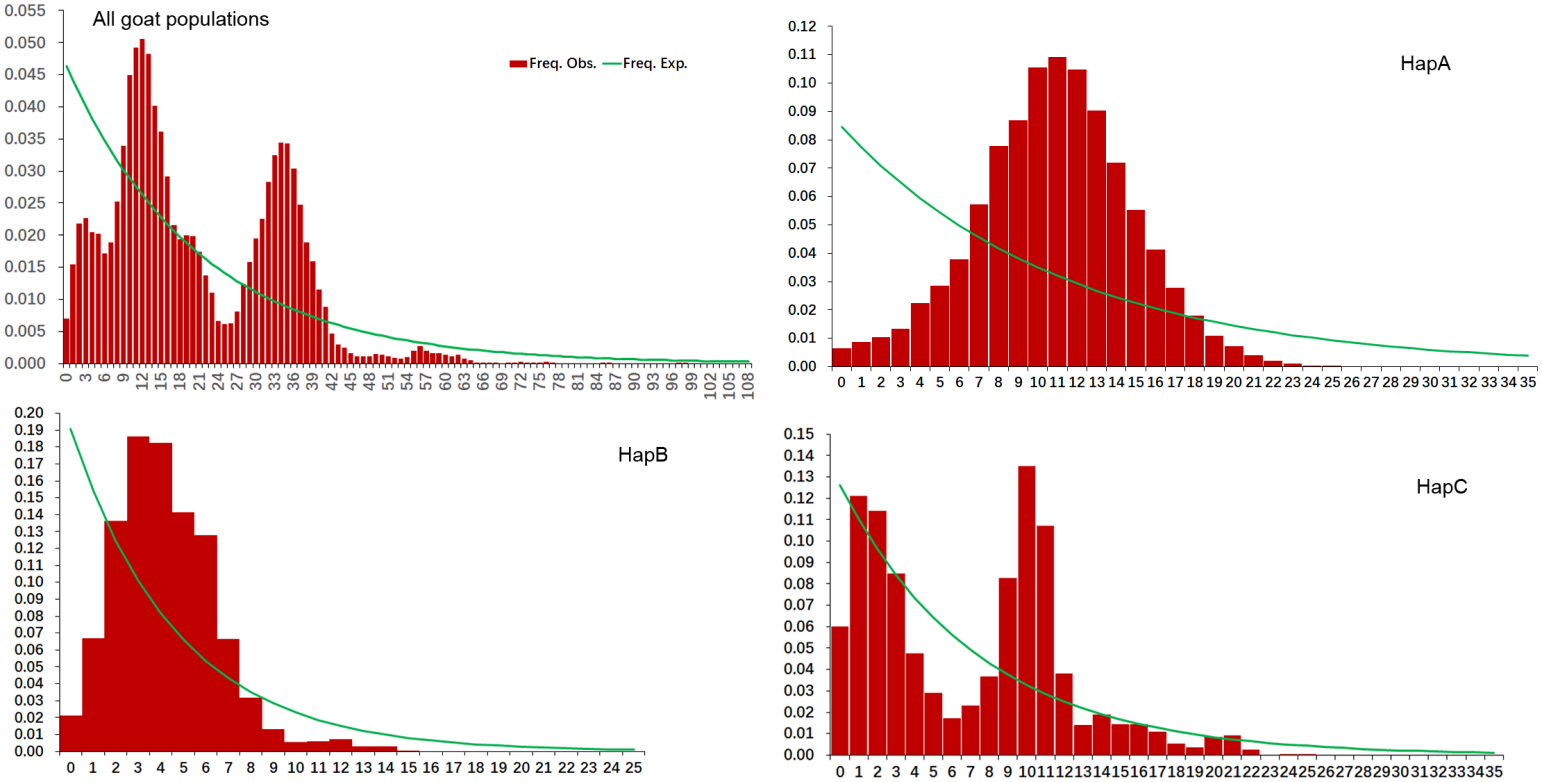
Mismatch distribution graphs for the 30 goat populations in China and Haplogroups A, B, and C analyzed in this study. The *x*‐axis shows the number of pairwise differences, and *y*‐axis shows the frequency of the pairwise comparisons.

Past population expansion events were inferred based on the patterns of mismatch distributions (Figure 8) and neutrality test estimates (Table 4). The mismatch distributions showed ragged and bimodal patterns for each ecogroup and haplogroup, and the observed pattern did not deviate significantly from that expected under a null hypothesis model of either spatial or demographic expansion. Tajima's D values for the goat ecogroups were not significantly different from zero for all populations, except for NYR (*p* < .05). Fu's *F*_s_ estimates for the goat ecogroups were negative and statistically significant. Tajima's *D* values were not significant for the haplogroups, and only the Fu's *F*_s_ estimate for HapA was significant. Furthermore, SSD and Happending's raggedness index (*r*) were determined to ascertain the goodness of fit of the mismatch distributions that varied among groups. The estimates for SSD and raggedness index values were positive and non‐significant for all groups, except for NYR and HapB. These results imply that the goats in the four ecogroups underwent different degrees of population expansion with HapA over time.

**TABLE 4:** Population demographic parameters estimated from the ecogroups.

Group	SWG	SCG	NYR	IBG	HapA	HapB	HapC
*N*	109	130	60	30	133	98	67
SSD, DE	0.005849	0.006452	0.007723*	0.013533	0.002602	0.033848**	0.022194
SSD, SE	0.008940	0.009913	0.008870	0.014257	0.003399	0.035360	0.019715
Raggedness index (*r*)	0.001977	0.002939	0.008368	0.011029	0.002228	0.010518	0.015002
Tajima's *D*	−0.650	−0.406	−1.623*	−1.075	−1.295	1.402	−0.502
Fu's *F*_s_	−23.898**	−23.931**	−20.381**	−7.828*	−23.836**	−5.474	−2.132

Abbreviations: DE, demographic expansion; *N*, sample size; SE, spatial expansion; SSD, sum of squared deviations.

*p < .05, **p < .01.

The genetic differentiation among the ecogroups was significant (Chi^2^ = 910.951, *p* < .001), and the estimated gene flow (Nm) was 2.17 (*F*_st_ = 0.10329). There was low gene flow between the ecogroups, of which gene flow between SCG and the groups NYR and IBG (*F*_st_ = 0.15107 and 0.13113) was lower than that between SCG and SWG (*F*_st_ = 0.08392), whereas the gene flow between IBG and the groups SWG and NYR (*F*_st_ = 0.05390 and 0.06562) was higher than that between SWG and NYR (*F*_st_ = 0.13926).

## 4 DISCUSSION

This study is an assessment of the genetic diversity, population structure, haplotype profile, and matriarchal ancestry of goat populations mainly from agro‐ecological zones in China where indigenous goats are reared, and provides insight into the genetic history of these goat populations based on mtDNA D‐loop sequences. Previous studies that investigated genetic diversity using AFLP (Liu et al., 2014) and microsatellite (E et al., 2019) markers and maternal origin (Li et al., 2021; Liu et al., 2006) only considered a few populations representing a few agro‐ecological zones. In this study, we included 329 goats of 25 native Chinese breeds and five introduced breeds from four ecogroups in both the private and public breeding sectors. The data of introduced goat breeds that contributed to the genetic improvement of native goats were included to evaluate their impact on Chinese native goat breeds and to determine the maternal origin of the native breeds.

China's geographical ecology is complex and diverse and is the basis of livestock genetic diversity (Cheng et al., 2020; Wang et al., 2020). The results obtained in the current study provide deeper insights into the genetic diversity of the analyzed breeds, which showed greater genetic diversity and MER for each site in the mtDNA D‐loop in SCG. SCG has become a habitat for goat populations with unique geographical isolation conditions due to the intersections of mountains and rivers; This region is isolated from SWG by the Daba, Wushan, and Wuling Mountains, and NYR by the Yangtze River and Dabie Mountains. Although a large proportion of genetic variation was observed from within the variety (76.7%, *p* < .001), we obtained 7.95% (*p* = .001) of the diversity among ecogroups in the population structure analysis, presenting regional differentiation of haplotypes, and 15.35% (*p* < .001) among breeds within ecogroups, indicating a stronger phylogeographic structure in the analyzed Chinese goats than previous studies (Luikart et al., 2001; Zhong et al., 2013). However, there is insufficient evidence that the genetic diversity of black goats is higher than that of white goats (Zhong et al., 2013).

Using the available goat mtDNA haplogroup classification system (Naderi et al., 2007), we identified three haplogroups (A, B, and C) among the Chinese native goat populations. The predominance of HapA in Chinese goat populations with the highest frequencies in SWG and NYR (0.53–0.69) was expected, as it is the most diverse and oldest population; HapA's wide distribution was consistent with the world (IBG, 0.7667) scenario described in previous studies (Naderi et al., 2007) in all goat breeds in the world. Interestingly, the frequency of HapA in the SCG (<0.09) was very low, and the expected haplotype flow was not observed. In contrast, HapB was mostly found in SCG (0.5538) and was distributed to NYR (0.2667) which is 1000 km away, whereas it was rarely distributed in SWG (<0.08), which is adjacent to SCG, and IBG (<0.07). These results indicate that HapB may belong to the original haplotype group of native Chinese goats and that the effect of human migration or commercial trade (Fernández et al., 2006; Nguluma et al., 2021) on haplotype distribution has been weak.

Analysis of animal kinship and ancestry and estimation of population purity and protection have been performed on cattle and sheep populations (Ben Sassi‐Zaidy et al., 2022; Zheng et al., 2020). According to the assessment of the investigated population in Figure 6, the higher RDA of A8 in SCG may be evidence for TapB as the maternal ancestral haplotype; However, its distribution in IBG is 0 and its proportion in SWG (4.59%) is low. From the population demographic parameters estimated from the analysis of the complete mtDNA D‐loop, Fu's *F*_s_ values of the HapB and HapC haplogroups were negative but not statistically significant, indicating no population expansion.

## 5 CONCLUSIONS

Native Chinese goat breeds were mainly divided into three haplogroups: HapA, HapB, and HapC. HapA, HapB, and HapC were associated with five, one, and two maternal ancestors, respectively. The goat populations expanded in the different ecoregions, indicating that native Chinese goats originated from two to three matrilineal ancestors and had no homology with imported goats.

## AUTHOR CONTRIBUTIONS

**Wenjuan Qin:** Conceptualization (lead); data curation (supporting); investigation (equal); methodology (equal); project administration (equal); resources (equal); writing – original draft (lead); writing – review and editing (equal). **Daosong Chen:** Formal analysis (equal); investigation (equal). **Panpan Guo:** Formal analysis (equal); investigation (equal). **Lixing Hu:** Formal analysis (equal); investigation (equal). **Xiaodong Zheng:** Data curation (equal); software (supporting). **Jin Cheng:** Conceptualization (equal); investigation (equal); methodology (equal); project administration (equal); resources (equal); supervision (equal); writing – original draft (equal). **Hongquan Chen:** Conceptualization (equal); data curation (equal); funding acquisition (lead); investigation (equal); methodology (equal); project administration (equal); visualization (equal); writing – review and editing (supporting).

## CONFLICT OF INTEREST STATEMENT

No potential conflict of interest was reported by the authors.

## Supporting information


Table S1

Appendix S1

## Data Availability

All sequences can be seen on GenBank, with accessions provided in Table S1.
