# Supplementary material for: Ecogroups and maternal haplogroups reveal the ancestral origin of native Chinese goat populations based on the variation of mtDNA D‐loop sequences
Source: Ecol Evol. 2023 Aug 7;13(8):e10382. doi: 10.1002/ece3.10382 (PMC10405232; doi:10.1002/ece3.10382)
Supplement: Supplementary file 1 — Table S1 [file ECE3-13-e10382-s002.docx]

Table S1 GenBank accession numbers of analyzed goat samples

| Breed/Population | GenBank accession | Breed/Population | GenBank accession |
| --- | --- | --- | --- |
| Qianqiu | OP921592-OP921612 | Longlinghuang | KM464514-KM464527 |
| Huanghuai | DQ121550-DQ121563 | Longlinhei | KM586101-KM586114 |
| Matou | DQ121577-DQ121590 | Yunlinghei | KM464542-KM464553 |
| Chuandongbai | AY860880-AY860884 | Beichuanbai | AY860871-AY860874 |
| Banjiao | DQ121491-DQ121505 | Tibet | AY860934-AY860942 |
| Guizhoubai | DQ121535-DQ121549 | Taihang | GQ141250-GQ141254 |
| Liaoning C | AY860909- AY860913 | Daiyunhei | KM586115-KM586129 |
| Guishanhei | KM464500,2-5,7-13 | Yangchengbai | GQ141255-GQ141258 |
| Shannanbai | DQ121604-18; KP195268 | Leizhouhei | DQ121564-76; KM586087-100 |
| Qianbeima | HQ199102-3,39-43,88-90 | Yudongbai | HQ199125-31; HQ199184-5 |
| Inner Mongolia C | DQ188883 | Haimen | GU223571 |
| Jiningqing | KP677510 | Boer | GQ141235-7; KJ420458 |
| Nubian | KJ420452,63,84 | Saanen | KJ420459-61,67,71,73-75,77,81,87 |
| Toggenburg | KJ420447,58,74,77,79,85; KY564252 | Angora | AB162203,AB110561-2;  GQ141233-4 |
| Chengduma | DQ121506,DQ121514-20; KM586074-82,84-86 | Dazu | HQ199094-5, HQ199111,  HQ199120-3, HQ199173-9 |
